# Activation of Abl1 Kinase Explored Using Well-Tempered Metadynamics Simulations on an Essential Dynamics Sampled Path

**DOI:** 10.1021/acs.jctc.1c00505

**Published:** 2021-10-14

**Authors:** Baswanth Oruganti, Ran Friedman

**Affiliations:** Department of Chemistry and Biomedical Sciences, Faculty of Health and Life Sciences, Linnæus University, 391 82 Kalmar, Sweden

## Abstract

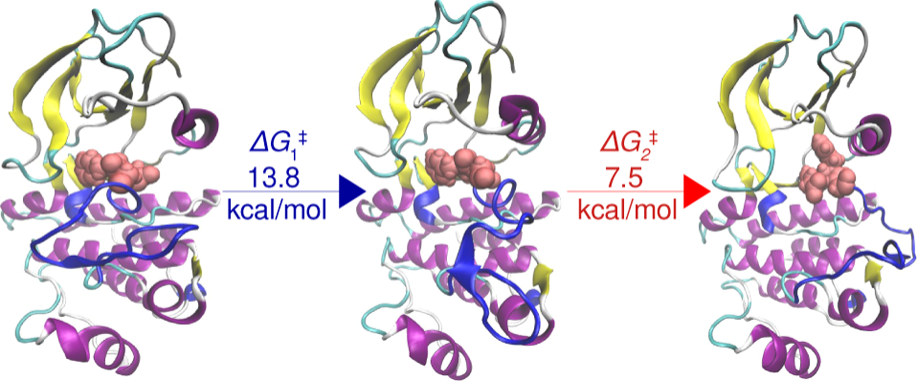

Well-tempered metadynamics (wT-metaD) simulations using path collective variables (CVs) have been successfully applied in recent years to explore conformational transitions in protein kinases and other biomolecular systems. While this methodology has the advantage of describing the transitions with a limited number of predefined path CVs, it requires as an input a reference path connecting the initial and target states of the system. It is desirable to automate the path generation using approaches that do not rely on the choice of geometric CVs to describe the transition of interest. To this end, we developed an approach that couples essential dynamics sampling with wT-metaD simulations. We used this newly developed procedure to explore the activation mechanism of Abl1 kinase and compute the associated free energy barriers. Through these simulations, we identified a three-step mechanism for the activation that involved two metastable intermediates that possessed a partially open activation loop and differed primarily in the “in” or “out” conformation of the aspartate residue of the DFG motif. One of these states is similar to a conformation that was detected in previous spectroscopic studies of Abl1 kinase, albeit its mechanistic role in the activation was hitherto not well understood. The present study establishes its intermediary role in the activation and predicts a rate-determining free energy barrier of 13.8 kcal/mol that is in good agreement with previous experimental and computational estimates. Overall, our study demonstrates the usability of essential dynamics sampling as a path CV in wT-metaD to conveniently study conformational transitions and accurately calculate the associated barriers.

## 1 Introduction

Some of the key challenges in the targeted therapy of cancer include identifying protein targets that can be selectively inhibited, accounting for the conformational variability in the protein targets and understanding the molecular basis for treatment-induced drug resistance. Addressing these challenges requires detailed mechanistic insights into the conformational transitions of proteins in their native and mutant forms by means of experimental and computational methodologies. While spectroscopic and crystallographic studies offer valuable insights in this regard, they are oftentimes tedious and highly resource-intensive. On the computational side, modeling these transitions using conventional molecular dynamics (MD) simulations is often unfeasible due to their relatively long timescales. However, enhanced sampling methods such as essential dynamics sampling (EDS),^1−3^ umbrella sampling,^4^ metadynamics,^5−8^ path-sampling approaches,^9^ and Markov state models^10,11^ were successfully used to explore such long-timescale processes in several proteins. Additionally, a hybrid model, which employed explicit solvent to propagate conformational dynamics and implicit solvent to compute free energies, coupled with a pH replica exchange scheme was used recently to map the conformational landscape of a tyrosine kinase protein.^12^

Protein kinases are drug targets in many cancers and in other diseases. As protein kinases typically adopt two distinct conformational states (active and inactive), they serve as excellent prototypes for testing new computational methodologies to investigate conformational transitions. In a recent study exploring the mechanism of activation of the FLT3 kinase, a combination of EDS and implicit solvent MD simulations was employed by our group to estimate the associated Gibbs free energy barriers.^3^ While EDS provides details on the transitions and reveals metastable intermediates that can be targeted by structure-based drug design^3^ or shed light on the biology of the system,^1^ associated transition energies cannot be obtained *solely* by EDS. For this reason, the Gibbs free energies were estimated in the aforementioned study by performing multiple implicit solvent MD simulations on conformations obtained from EDS. However, interactions of the protein with solvent vary with protein conformation, which cannot be fully accounted for in implicit solvent. Moreover, implicit solvent MD simulations are not available in newer versions of the popular computational packages such as GROMACS,^13−15^ which is, to our knowledge, the only package that implements EDS. As such, it is desirable to couple EDS to a sampling method that can be utilized for Gibbs free-energy calculations in explicit solvent.

Using well-tempered metadynamics (wT-metaD) simulations with path collective variables (CVs) is a well-known enhanced sampling approach that has been successfully employed to identify metastable states and estimate free energy profiles of conformational transitions in protein kinases such as cyclin-dependent kinase 5^5^ and adenylate kinase.^7^ Furthermore, this method has also been applied to compute the binding free energy barriers of imatinib to the wild-type Abelson tyrosine kinase (Abl1) and to its T315I “gatekeeper” mutant.^6^ Although this approach has the advantage of limiting the number of CVs and bypasses the problem of finding CVs (such as interatomic distances or dihedrals) that can describe the conformational transition of interest, it requires as an input a reference path connecting the initial and target states. Such a path can be generated, for example, by means of a preliminary wT-metaD run using geometric CVs^6^ or by a geometric interpolation between the initial and target states.^5,7^ The success of the former method still relies on the choice of CVs, while the latter method may generate some unphysical intermediate states. On the other hand, EDS uses principal components (PCs) to represent large-amplitude collective motions of the protein and can potentially circumvent these limitations. This work aimed to couple EDS and wT-metaD simulations to explore the mechanism of activation of the Abl1 kinase and compute the associated free energy barriers.

Abl1 is a nonreceptor type kinase whose activity is tightly regulated by phosphorylation of a tyrosine (Tyr^393^) residue. A common feature in cancers such as chronic myeloid leukaemia (CML) and rare cases of acute lymphocytic leukaemias is the constitutively active Abl1 enzyme, resulting from a genetic fusion of the Abl1 gene in chromosome 9 with the breakpoint cluster region (BCR) gene in chromosome 22. This leads to the creation of the BCR–Abl1 fusion gene and the so-called Philadelphia chromosome (Ph). Impairing the activity of the BCR–Abl1 chimeric protein by means of small molecule drugs such as imatinib (Gleevec) selectively inhibits the proliferation of CML cells and is now a well-established targeted therapy in the treatment of CML.^16^ However, a key challenge in this treatment has been addressing the drug resistance in patients due to treatment-induced mutations in the protein.^17−20^

**Figure 1:**
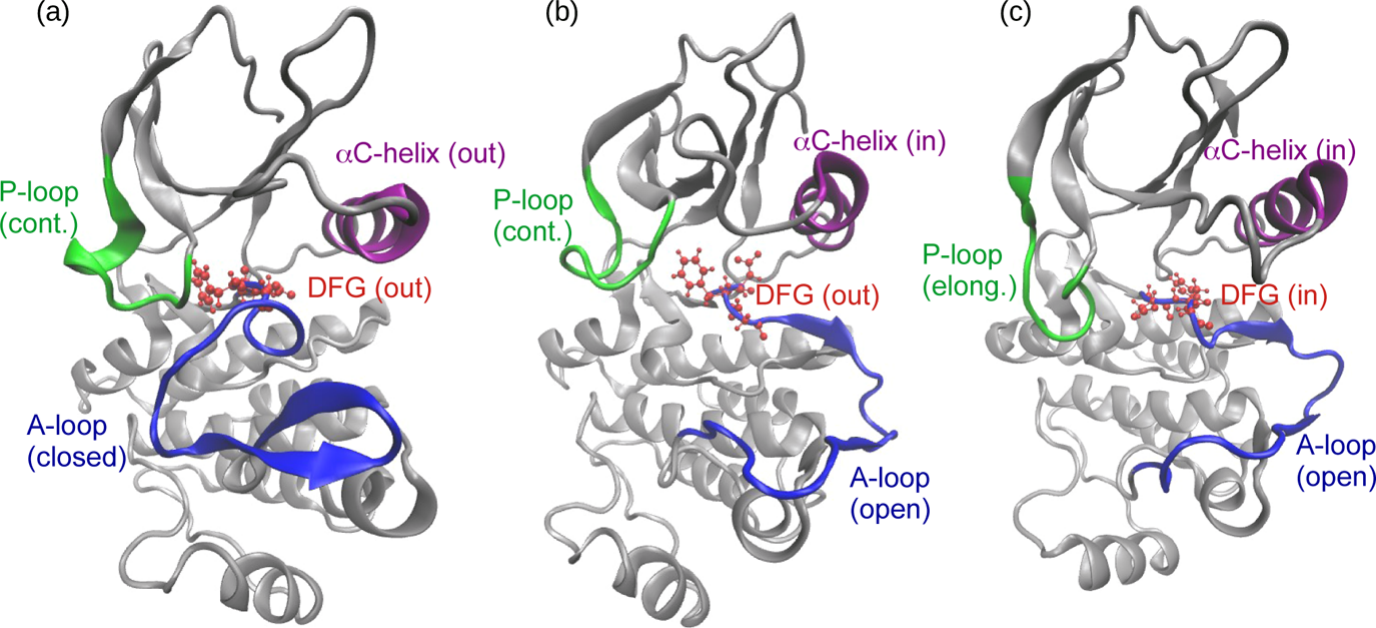
Structures of the inactive (a), inactive-2 (b), and active (c) states of Abl1 kinase. The four key structural elements, namely, the A-loop, the αC-helix, the DFG motif, and the P-loop are labeled.

The activation of Abl1 kinase involves a conformational transition from an inactive state to an active state. The difference between the states is usually manifested in four structural elements: the activation loop (A-loop), the αC-helix, the Asp–Phe–Gly (DFG) motif, and the phosphate-binding loop (P-loop); see Figure 1. Typically, in the inactive state, the A-loop and αC-helix adopt so-called “closed” and “out” conformations, respectively, which effectively block the access of substrates to the binding site. In the native protein, phosphorylation triggers the activation by transforming the A-loop and αC-helix from the “closed” and “out” conformations in the inactive state to the “open” and “in” conformations in the active state, as illustrated in Figure 1. Furthermore, the two states differ also in the “out” (inactive) or “in” (active) orientation of the Asp residue of the DFG motif and the contracted (inactive) or elongated (active) nature of the P-loop, as shown in Figure 1. Interestingly, a recent spectroscopic study of Abl1 kinase inactivation detected another “inactive” state that mediates the conformational transition and in which the DFG motif is flipped by 180° with respect to the active state, while the A-loop remains in the open conformation.^21^ This intermediate state is hereafter referred to as the inactive-2 state (Figure 1b) to distinguish it from the aforementioned inactive state (see Figure 1a).

In this study, we propose a composite computational strategy that combines EDS^22^ with wT-metaD^23^ simulations. This method was used to explore the mechanism for the activation of wild-type Abl1 kinase as a test case. Specifically, by employing a path sampled from a short EDS as one path CV^24^ and the distance from the input EDS path as another in wT-metaD simulations (as further detailed in theComputational Methods section), this method offers enhanced variational flexibility by allowing for the possibility of exploring activation pathways that could be more favorable than the input EDS path. Through these calculations, we identified a three-step mechanism for the activation that involves two intermediate states with a partially unfolded A-loop, which differ mainly in the DFG conformation. Finally, in this study, we have also investigated the applicability of this computational strategy in modeling the activation dynamics of the phosphorylated Abl1 kinase.

## 2 Computational Methods

All MD and EDS simulations were carried out using the GROMACS program (v2018.4),^13,14^ whereas wT-metaD^23^ simulations were performed using the PLUMED^25,26^ version of GROMACS, v2019.5-plumed. The CHARMM36^27^ force field was used for solute atoms, and the TIP3P^28^ model was employed for water molecules. A cutoff distance of 1.2 nm was used to compute van der Waals and Coulomb interactions. The long-range electrostatic interactions were modeled using the PME method.^29,30^ The LINCS algorithm^31^ was used to constrain bonds involving hydrogen atoms, and the SETTLE algorithm^32^ was employed to constrain rigid water molecules. The simulations were performed with the NPT ensemble at *T* = 300 K, using the velocity rescaling thermostat^33^ (0.1 ps), and at *P* = 1 bar, kept constant by the Berendsen barostat^34^ for the equilibration and by the Parrinello–Rahman barostat^35^ for the production runs. The solvent accessible surface area (SASA) was calculated with the *gmx sasa*(36) tool, and hydrogen bonds were calculated using the *gmx hbond* tool in GROMACS.

### 2.1 MD Simulations

In the case of both the non-phosphorylated (non-phos) and phosphorylated (phos) systems, the starting configurations of Abl1 kinase used in the MD simulations were the crystal structures obtained from the Protein Data Bank (PDB)^37^ files: PDB ID 2GQG(38) and PDB ID 2HYY,^39^ solved by X-ray diffraction at 2.4 and 2.1 Å resolutions, respectively. While 2GQG corresponds to the active state of a phos system, 2HYY corresponds to the inactive state of a non-phos system. The corresponding non-phos structure in the former case and the phos structure in the latter case were then generated from the 2GQG and 2HYY PDB files, respectively, using PyMOL. Prior to running MD simulations, energy minimization was performed on all the structures for 50,000 steps using the steepest decent algorithm. Subsequently, a short (20 ps) MD simulation was performed with positional restraints on all heavy (non-hydrogen) atoms of the protein in order to equilibrate the water molecules surrounding the protein. Next, a 10 ns equilibration run was performed after removing the restraints. Production MD simulations of 50 ns were then performed at constant pressure (1 bar) and temperature (300 K) on both the active and inactive states by running five different trajectories for the non-phos and phos systems. Coordinates and energies were saved every 10 ps (except for one trajectory where the values were saved every 1 ps).

### 2.2 Analysis of the MD Simulations

Cluster analysis, based on the Cα atoms of the protein, was performed on all the MD simulations of both the non-phos and phos systems using the algorithm developed by Daura and co-workers^40^ with a cut-off of 0.15 nm. Subsequently, to identify collective modes of fluctuations, covariance analysis was performed using the *gmx covar* utility in GROMACS, taking into account all the heavy atoms and using only a 10 ns fragment of the trajectory in which the protein remained in the same cluster. The reason for this choice is to exclude eigenvectors associated with random diffusion due to motion of the system between two different clusters. The eigenvectors obtained from the diagonalization of the covariance matrix are called PCs of which the dominant components represent large-amplitude collective motions associated with conformational changes.

### 2.3 Essential Dynamics Sampling

To generate a reference path for wT-metaD simulations, EDS^2,22,41,42^ was used, where a trial generated by the normal MD simulation was accepted if the root-mean-square deviation (rmsd) to the target active structure diminished. When the simulated structure moves away from the active structure, the coordinates and velocities were projected onto the essential subspace of the active structure. In principle, this strategy can facilitate the system to reach the active state in a short span of time, but this is not guaranteed. The EDS algorithm was used as implemented in GROMACS. The EDS input file was generated using the *gmx make_edi* utility. The simulations were run for 500 ps for both the non-phos and phos systems. Coordinates and energies were saved every 1 ps.

### 2.4 wT-metaD Simulations Using Path CVs

In metadynamics simulations,^23,24,43−46^ a history-dependent Gaussian bias potential as a function of a finite set of CVs is added to the Hamiltonian of the system. The added potential fills the underlying free energy basins and enables an efficient exploration of the free energy space. In wT-metaD simulations,^23^ the Gaussian height is decreased during the simulation to avoid overfilling of the free energy basins and to ensure the convergence of the final bias potential to the actual free energy (within a constant). Large conformational transitions such as between inactive and active states of kinases (Figure 1a,c) are challenging to simulate even with wT-metaD simulations. In order to describe conformational transitions of kinases, two path CVs, *s*(*R*) and *z*(*R*) were found to be useful.^5,7,24^ The former CV describes the progress of the reaction along a predefined reference path connecting the initial and target states, and the latter describes the distance from the reference path. These CVs are defined as follows
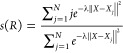
1
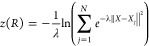
2where *N* is the number of snapshots in the reference path, and ∥*X* – *X*_*j*_∥^2^ is the mean squared displacement (MSD) between an instantaneous configuration *X* and a configuration *X*_*j*_ along the reference path after optimal alignment. λ is proportional to the inverse of the MSD between the consecutive frames in the path and, based on previous recommendations,^5,7,24^ was taken as
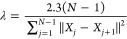
3

In this work, a path sampled from the EDS simulation was used as the reference path CV *s*(*R*). The sampling was performed by choosing 113 and 99 frames from the EDS path for the non-phos and phos systems, respectively. The frames were ordered, so that the rmsd between the successive frames remained similar, between 0.44 and 0.60 Å, as detailed further in the Results and Discussion section. In order to facilitate the comparison between the non-phos and phos systems, where different numbers of frames were used, normalized *ŝ*(*R*) was used instead of *s*(*R*). As dictated by the convergence of resulting Gibbs free energy profiles, simulations were run for 80 ns for the non-phos system and 120 ns for the phos system. Gaussians were deposited every 500 steps with the initial height set to 0.5 kJ/mol, reducing the Gaussian height using a bias factor of 30. The widths of Gaussians in units of *s*(*R*) and *z*(*R*) were set to 2.0 and 0.005 Å^2^, respectively. To limit the exploration of the phase space with very high energies, a restraining force of 10^4^ kJ ·mol^–1^ Å^–2^ was applied when *z*(*R*) exceeded 3.0 and 5.0 Å^2^ for the non-phos and phos systems, respectively. A larger boundary on the *z*(*R*) value was employed for the latter system because the activation was not observed within the simulation time when smaller values had been used.

## 3 Results and Discussion

The inactive → active transitions of the non-phos and phos systems were explored in the following way. First, five conventional MD trajectories of 50 ns were run for both the inactive and active states. Starting from the inactive state and using the essential subspace of the target active state, a short EDS simulation of 500 ps was performed using each of the trajectories. Subsequently, one representative EDS simulation was used to sample configurations from the path connecting the starting state to the target state. The sampled path and the deviation from it were then used as path CVs for performing wT-metaD simulations.

### 3.1 Elucidation of Activation Pathways with EDS

**Figure 2:**
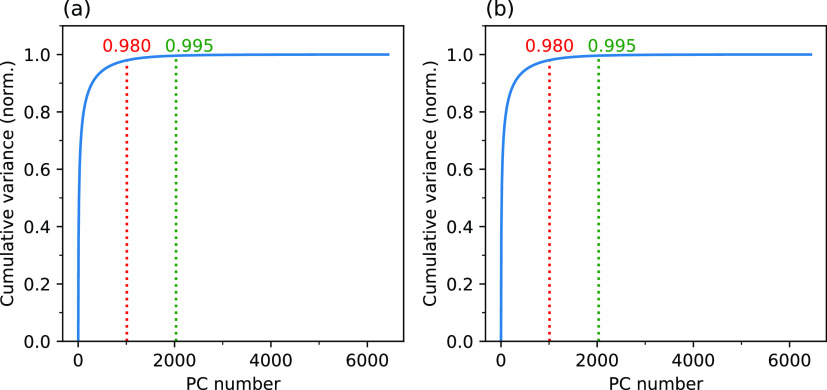
Normalized cumulative variance as a function of the number of PCs for one of the trajectories of the active state of the non-phos (a) and phos (b) systems.

Covariance analysis was performed on all the five MD trajectories by choosing a 10 ns fragment of each trajectory in which the system remained in the same configuration, as indicated by cluster analysis. In order to identify the essential subspace of the protein that spans large amplitude collective motions associated with the inactive → active transition, the normalized cumulative variance was plotted against the number of PCs of the target active state for one of the trajectories (for which the coordinates were saved at 1 ps interval). As can be seen from Figure 2, the first ∼1000 (∼2000) eigenvectors constituted 98% (99.5%) of the total protein fluctuation. Furthermore, it is notable that 2000 PCs constituted a substantial ∼30% of the total eigenvector space.

**Figure 3:**
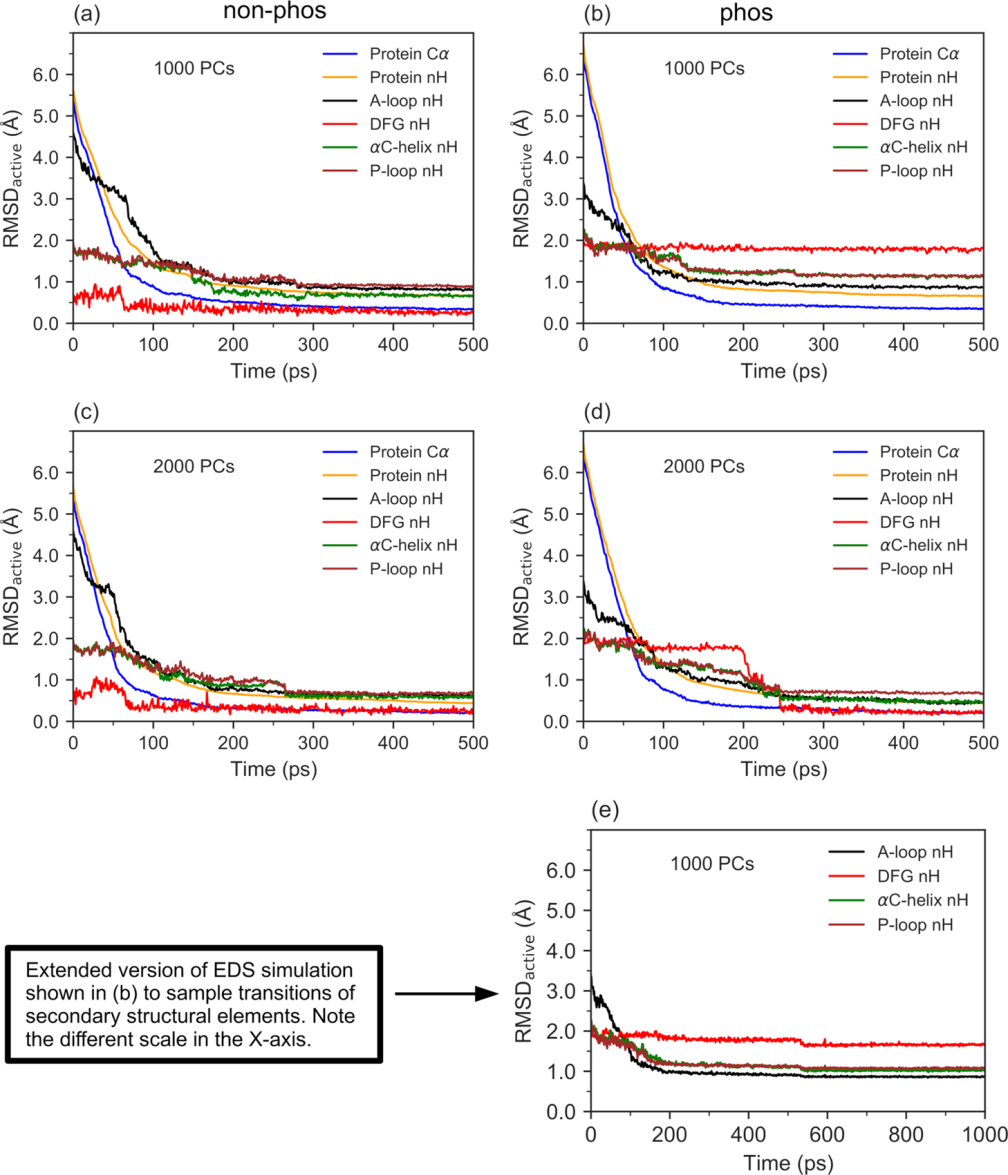
Variation in rmsd relative to the target active state (rmsd_active_) of the non-phos (a) and (c) and the phos (b), (d), and (e) systems during EDS simulations. Before each type of rmsd calculation, EDS structures were fitted to the active state using either Cα (for Cα-rmsd) or non-hydrogen (for nH-rmsd) atoms of only the structural element under consideration.

In order to test how the choice of size of the essential subspace influenced the EDS of the protein, two different subspaces, one with 1000 PCs and the other with 2000 PCs, were used for one of the five EDS simulations, whereas only 1000 PCs were employed for the other four simulations. The results obtained from the former simulation are presented in Figure 3, while the results from the latter four EDS simulations are given in Figure S1 of the Supporting Information.

In the non-phos system, the rmsd calculated for all non-hydrogen atoms (nH) of the protein decreased from ∼5.6 Å at the beginning of the simulation to ∼0.7 Å at 300 ps. In the case of Cα atoms of the protein, the rmsd decreased from ∼5.4 to ∼0.4 Å. A similar trend is also reflected in the nH atoms of the individual secondary structural elements, namely, the A-loop, DFG motif, αC-helix, and P-loop. Furthermore, the trends seem to be relatively independent of the choice of size of the essential subspace.

In the phos system, the nH and Cα rmsd decreased from ∼6.5 to ∼0.7 and ∼0.4 Å, respectively, in about 300 ps. However, in contrast to the non-phos system, these trends are not well reflected in individual structural elements for the simulation in which the essential subspace constitutes only 1000 PCs. Specifically, the rmsd variations in the nH atoms of the DFG motif, αC-helix, and P-loop were all smaller by 0.6–1.6 Å for the essential subspace constituting 2000 PCs compared to those for the subspace of 1000 PCs. Apparently, even if 1000 PCs cover 98% of the conformational space of the phos system, small fluctuations appear to be crucial for the rmsd convergence of the secondary structural elements. In order to examine if extending the EDS simulation time rather than increasing the size of the essential subspace improves the rmsd convergence of the secondary structural elements, a new EDS simulation was run for a time of 1000 ps using only 1000 PCs. As can be seen from the results of the simulation shown in Figure 3e, rmsd values of all the structural elements remained essentially unaltered from 300 to 1000 ps, thereby emphasizing the redundancy of longer sampling times and the necessity of a larger subspace in capturing small-amplitude transitions, particularly the DFG flip. Thus, the dynamics of the phos system seems to be more intricately regulated as can be inferred from its sensitivity to the size of the essential subspace. While further increasing the size of the essential subspace up to 3000 PCs (account to ∼45% of the total number of PCs) may improve sampling of changes in the individual structural elements, it was not feasible due to memory constraints.

To sample a path from EDS simulations that connected inactive and active states, a single representative EDS simulation was considered in the case of the non-phos system. This is because all EDS simulations (left panels in Figures 3 and S1 of the Supporting Information) exhibited similar features in terms of successful completion of the transitions of the secondary structural elements by ∼300 ps, albeit the transitions occurred at different time points during this period. However, in the case of the phos system, the simulation run with 2000 PCs was employed as the simulations run with 1000 PCs could not capture well small-amplitude transitions of the secondary structural elements (right panels in Figures 3 and S1 of the Supporting Information). For both the non-phos and phos systems, only the Cα atoms of the protein were used in the path sampling. Two criteria were employed for sampling that are known to be essential for performing wT-metaD simulations with path CVs.^5,7,24^ First, the frames constituting the sampled path were selected, such that the rmsd in terms of protein Cα was similar between two selected consecutive frames, that is, 0.44–0.60 Å for both the non-phos and phos systems, as illustrated in Figure S2 of the Supporting Information. These rmsd values yielded λ values of 9.17 and 8.77 Å^–2^ for the non-phos and phos systems (eq 3). Second, as is evident from Figure S3 of the Supporting Information, the sampled frames are topologically consecutive, in that a given frame is closer to the target state (in terms of Cα rmsd) than all other frames preceding it.

### 3.2 Metadynamics Improve upon EDS Path

Considering the EDS path *s*(*R*) and distance from the path *z*(*R*) as the two path CVs as detailed in the Computational Methods section, wT-metaD simulations were performed to model the activation pathways and calculate the free energy profiles of both the non-phos and phos systems.

#### 3.2.1 Three-Step Activation Mechanism

**Figure 4:**
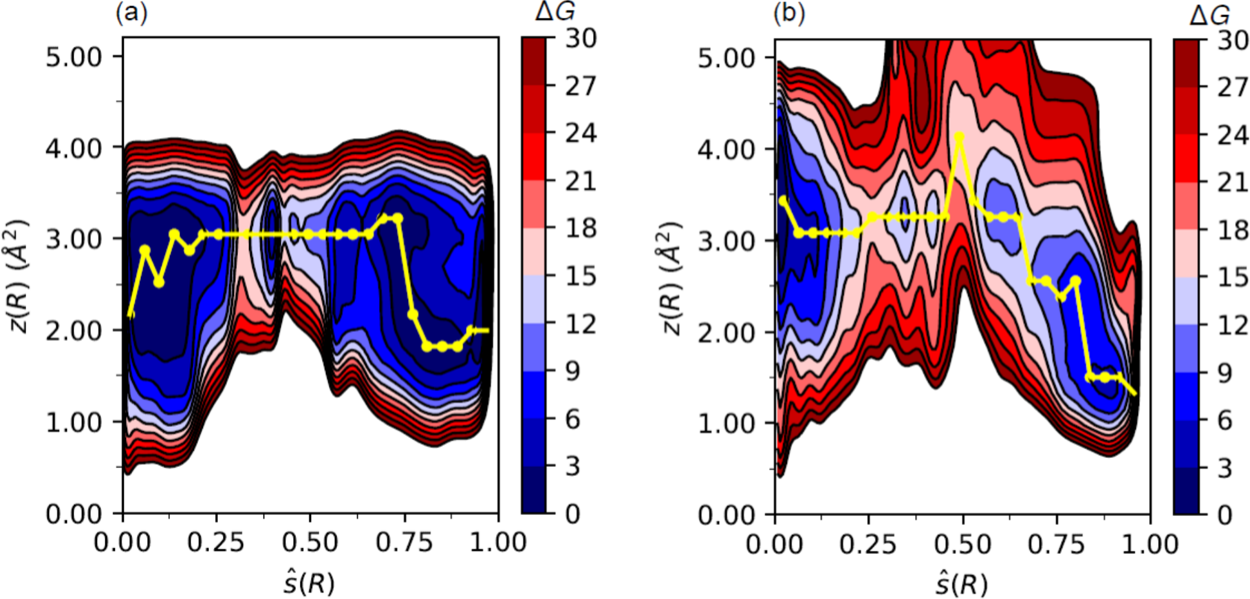
Activation free energy (Δ*G*, kcal/mol) profiles of the non-phos (a) and phos (b) systems as a function of the *ŝ*(*R*) and *z*(*R*) CVs obtained from wT-metaD simulations. MFEP for activation is shown in yellow color.

The free energy profiles of both the non-phos and phos systems as a function of the *ŝ*(*R*) and *z*(*R*) CVs obtained from wT-metaD simulations are shown in Figure 4a,b, respectively. For both systems, a minimum free energy path (MFEP) connecting the inactive and active states as a function of *ŝ*(*R*) was generated by integrating out *z*(*R*) as follows. The full configuration space of *ŝ*(*R*) was divided into 25 bins of equal width, and within the each bin, the value of *z*(*R*) that yielded the lowest free energy was considered.

Interestingly, it can be inferred from the MFEPs shown by yellow colored lines in Figure 4 that the minimum free energy basins at *s*(*R*) ≈ 0 and *s*(*R*) ≈ 1 corresponding, respectively, to the inactive and active states lie at small *z*(*R*) values of 1.0–3.5 Å^2^, while the intermediary configurations mediating the activation lie at relatively larger *z*(*R*) values of 3.0–4.2 Å^2^. As *z*(*R*) measures the distance from the input EDS path, the relatively larger *z*(*R*) values suggest that the path obtained from wT-metaD simulations is energetically more favorable than the input EDS path. This seems to be particularly true for the phos system, wherein the *z*(*R*) values for some of the intermediary configurations were larger by ∼1.0 Å^2^ than those of the non-phos system. This observation suggests that the dynamics of the phos system is not well captured by the EDS simulations and is also in line with the conclusion drawn from the EDS simulations that the dynamics of the phos system seems to be more intricately regulated.

**Figure 5:**
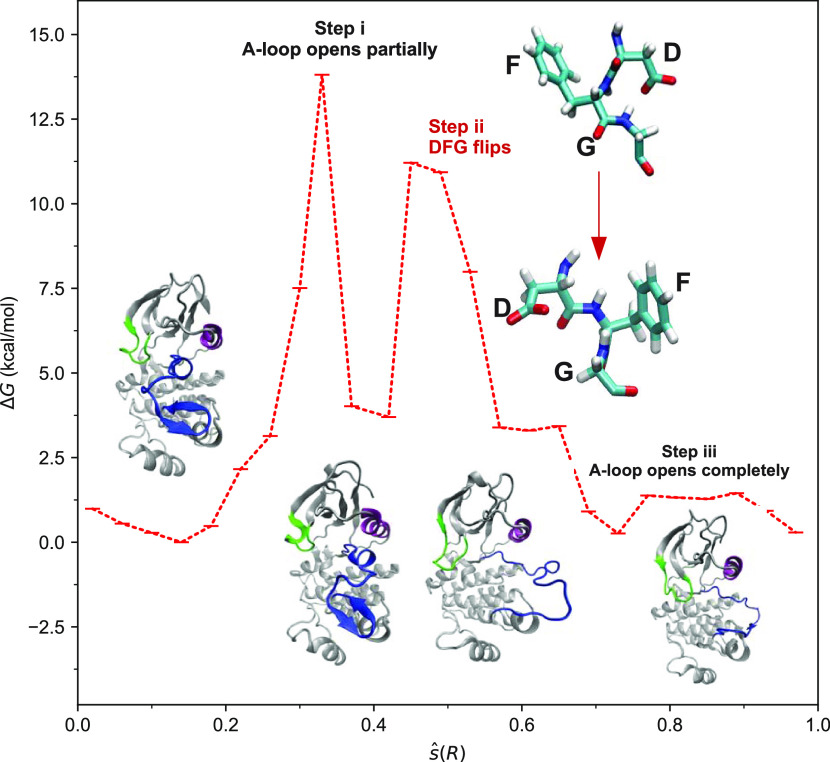
MFEP for the activation of the non-phos system as a function of the *ŝ*(*R*) CV obtained from wT-metaD simulations. Shown also are the structural changes involved in the opening of A-loop (in blue) and the DFG flip (in the inset).

**Figure 6:**
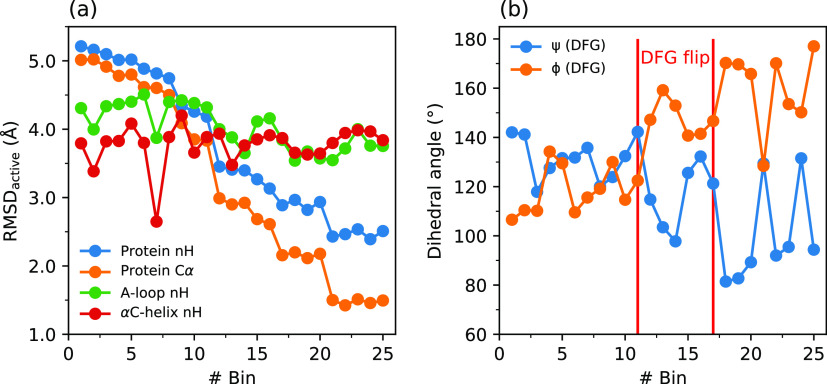
Variation in rmsd with respect to the target active state (a) and variation in the Ramachandran angles ψ and ϕ (b) during the MFEP for the activation of the non-phos system.

**Figure 7:**
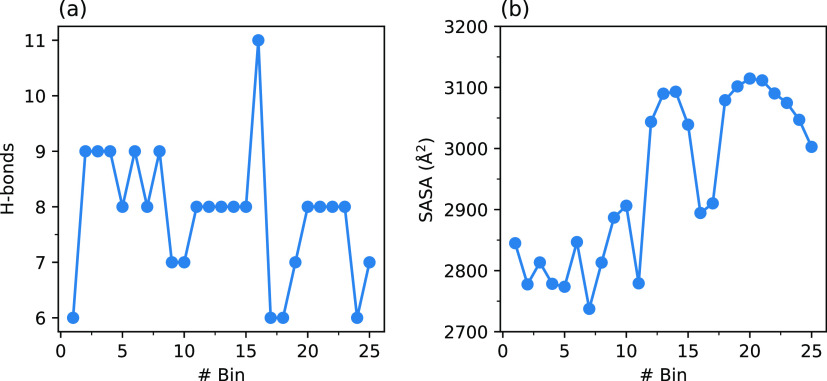
Variation in the number of hydrogen bonds between the A-loop and the solvent (a) and variation in the A-loop SASA (b) during the MFEP for the activation of the non-phos system.

The MFEP for the non-phos system as a function of the *ŝ*(*R*) CV is shown in Figure 5, and the corresponding path for the phos system is shown in Figure S4 of the Supporting Information. In the non-phos system, it can be noted from Figure 5 that the activation involves three distinct steps. In the first step, spanning *ŝ*(*R*) values from 0.0 to 0.4 and bins 1–11, structural changes occur mainly in the A-loop and αC-helix. This can be inferred from Figure 5 that presents snapshots from the MFEP and Figure 6a that shows the rmsd variations in the A-loop and the αC-helix with respect to those in the target active state. Specifically, partial unfolding of the A-loop occurs in this step that is accompanied by a concomitant increase in the number of hydrogen bonds between the A-loop and solvent molecules from 6 to 8 (see Figure 7a). This step occurs with a free energy barrier of 13.8 kcal/mol and is the rate-determining step of the activation. This value is in good agreement with previous spectroscopic^21^ and computational^47^ studies of Abl1 kinase that predicted activation barriers in the range of 14–17 kcal/mol under ambient conditions.

**Figure 8:**
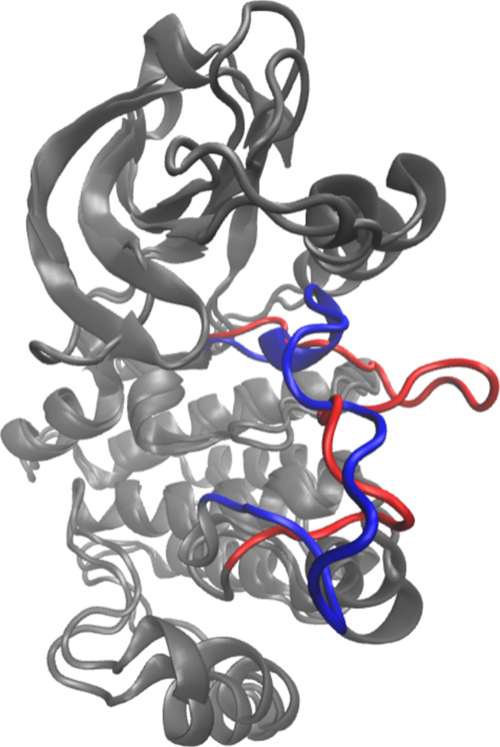
Superposition of the src-like crystal structure of Abl1 2G1T(48) (in red) with a representative structure (in blue) from bin 15 of the MFEP of the non-phos system. The A-loop is highlighted.

In the second step that spans bins 11–17 and *ŝ*(*R*) values from ∼0.4 to ∼0.7, structural changes occur mainly in the DFG motif with a large variation of up to ∼40° in the Ramachandran angles ψ and ϕ (see Figure S5 of the Supporting Information for their definitions) of Phe^382^ (Figure 6b). Specifically, there is a mutual exchange of positions between the side chain of Asp^381^ and the phenyl ring of Phe^382^, as depicted in the inset of Figure 5. This step occurs with a smaller free energy barrier of 7.5 kcal/mol and yields an src-like inactive state possessing a DFG-in conformation similar to a structure that was previously characterized by X-ray crystallographic measurements of Abl1 kinase (PDB ID: 2G1T)^48^ and implicated in computational studies.^11,47,49−51^ To visualize structural similarities, a representative configuration from bin 15 was superposed with the src-like inactive crystal structure 2G1T of Abl1 kinase (Figure 8). The full backbone rmsd of this structure with respect to the crystal structure was 3.2 Å. Furthermore, the computed barrier for the DFG flip is in agreement with the previous computational estimate of 7.0 kcal/mol for c-Abl kinase reported by Meng *et al.*, using umbrella sampling and string methods.^50^

In the third and final step that encompasses bins 17–25 and occurs essentially in a barrierless fashion, further changes in the Ramachandran angles occur accompanied by a complete unfolding of the A-loop as reflected in an increase in the A-loop SASA by up to 200 Å^2^ during this step (see Figure 7b). This step occurs with an exergonicity of 3.4 kcal/mol and completes the activation. Taking steps two and three together, the overall exergonicity for the DFG-out → DFG-in transition is 3.7 kcal/mol (*i.e.*, the DFG-in conformation is more favorable), which is well in line with the corresponding previous estimates of the DFG flip by Lovera *et al.* (4.0 kcal/mol)^51^ and Meng *et al.* (1.4 kcal/mol)^50^ in c-Abl kinase. Interestingly, it had previously been found that the protonation of the Asp^381^ residue flips the relative stabilities by making the DFG-out conformation to be more favorable by ∼1.0 kcal/mol.^50,52^

Overall, the mechanism involves two intermediates that differ in the extent of unfolding of the A-loop and the DFG orientation. These intermediates are structurally similar to five different previously reported conformations of Abl1 kinase,^11^ as can be seen from Table S1 of the Supporting Information, that quantifies structural similarity in terms of the full backbone rmsd. Furthermore, the sequence of changes in the A-loop and DFG motif during the *activation* is chronologically reversed relative to the previously identified mechanism of *inactivation* of Abl1 kinase by Narayan *et al.*(47) and Xie *et al.*(21)

In the MFEP for the phos system shown in Figure S4 of the Supporting Information, it can be noted that the highest barrier along the activation pathway is 16.1 kcal/mol. However, unlike the MFEP for the non-phos system that involves two metastable intermediate states that lie within ∼5 kcal/mol relative to the inactive state, only one such metastable state [*ŝ*(*R*) = 0.9 or bin 24] is observed for the phos system. Furthermore, the simulation protocol could not capture the DFG flip; as can seen from Figure S6 of the Supporting Information, the Ramachandran angles ψ and ϕ of the starting (bins 1–3) and final configurations (bins 23–25) were almost the same (see also the MFEP movies provided in the Supporting Information). This artifact resulted in unusually large free energies toward the end of the MFEP, as can be inferred from Figure S4 of the Supporting Information. Overall, simulations of the phos system reveal that the barriers are associated with the same transitions as in the non-phos system (except for the DFG-flip), but their heights are overestimated considering the fact that the phosphorylation of the Tyr^393^ residue of the Abl1 kinase stimulates the activation.^21,53^

**Figure 9:**
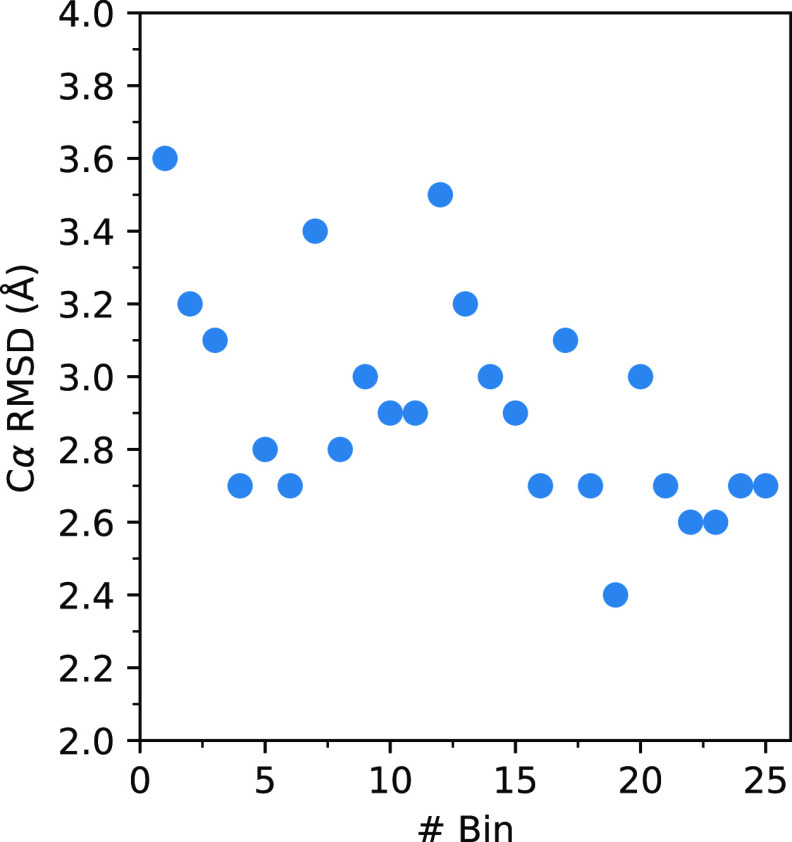
Variation in the Cα rmsd between the MFEPs of the non-phos and phos systems.

To understand the origin of overestimation of barriers for the phos system, the differences in MFEPs of the non-phos and phos system in terms of Cα rmsd are presented in Figure 9. Large rmsd values of 2.4–3.6 Å between the two MFEPs hint at possible alternate paths for the phos system, which are more proximate to the MFEP of the non-phos system but are not captured by the simulations. This could possibly be due to the 2000 PCs essential subspace being insufficient, although it captures ∼99.5% of the dynamics. Unfortunately, calculations with an even larger subspace of 3000 PCs were not possible because of memory constraints, as mentioned above in Section 3.1. Thus, it appears that the success of the proposed approach relies on the quality of the input EDS path in terms of its proximity to a MFEP as is commonly the case with any path CV in wT-metaD simulations.^24^ Besides the limitations associated with the essential subspace, another possible source of error could be inadequate sampling of structural changes stimulated by phosphorylation of the inactive state. Specifically, from a comparison of the location of the minimum corresponding to the inactive state for the non-phos system in Figure 5 (bin 4) and that for the phos system in Figure S4 of the Supporting Information (bin 1), it can be hypothesized that it is one of the conformations along the activation of the non-phos system that is phosphorylated, not directly the inactive state as was assumed when we ran the simulations. In other words, it is likely that an yet unidentified structural change (along the activation pathway) is required before Tyr^393^ can be phosphorylated.

#### 3.2.2 Error Estimation and Robustness of the Methodology

**Figure 10:**
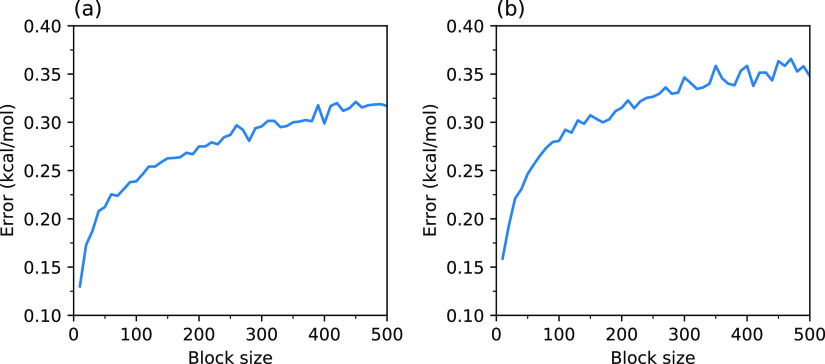
Mean error in the estimated free energies of the non-phos (a) and phos (b) systems as a function of the block size.

Having established a stepwise mechanism for the activation of non-phos and phos systems and computed the associated Gibbs free energy barriers, errors in the obtained free energies were estimated using an umbrella sampling-like reweighting approach as follows. To recover the original Boltzmann distribution from the biased distribution, unbiasing weights were calculated assuming a constant bias throughout the simulation and using the bias potential obtained at the end of the simulation as described by Branduardi *et al.*(54) The resulting data were discretized into 25 equally sized bins, and block averaging was performed in order to remove time correlations in the data. This was done using blocks of different sizes ranging from 10 to 500 in the steps of 10. The mean error over all the bins is plotted against the block size shown in Figure 10, and the errors within individual bins are shown in Table S2 of the Supporting Information. From Figure 10, it can be noted that the mean error converges to ∼0.3 kcal/mol for the non-phos system and ∼0.4 kcal/mol for the phos system. Considering the maximum error of ∼0.5 kcal/mol over all the bins as noted in Table S2 of the Supporting Information, the maximum error in the estimated free-energy barriers and relative energies amount to ∼1.0 kcal/mol for both the non-phos and phos systems.

**Figure 11:**
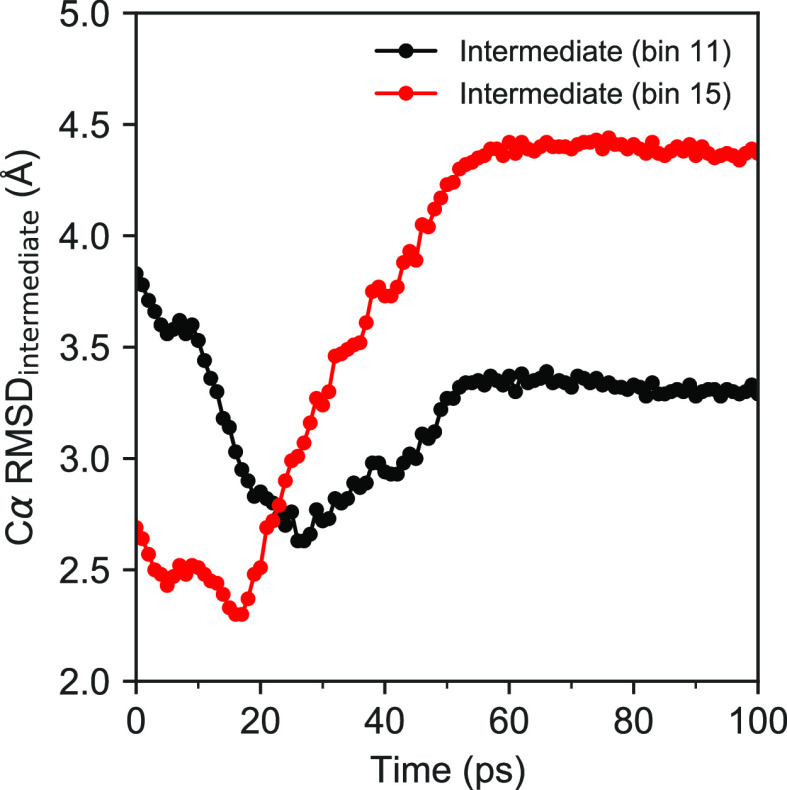
Cα rmsd between the two intermediates mediating the activation (bins 11 and 15 of MFEP in Figure 5) and the first 100 ps of the EDS simulation for the inactivation of the non-phos system.

Finally, to demonstrate that the identified mechanism for the activation is robust to the choice of the input EDS path, the two intermediates mediating the activation (see Figure 5) are compared in Figure 11 with structures from a new EDS simulation performed to model the inactivation rather than the activation of the non-phos system. Starting from the active state and employing the essential subspace of the target inactive state, the simulation was run for 500 ps using 1000 PCs. As one would expect, the chronology of the two intermediates in the EDS path for inactivation is reversed relative to the MFEP for activation. This can be inferred from Figure 11 by noting that the two intermediates corresponding to bins 11 and 15 of the MFEP are structurally closest in terms of Cα rmsd to the EDS snapshots at times 26 and 17 ps, respectively, with rmsd values of 2.6 and 2.3 Å.

## 4 Conclusions

A novel computational strategy that combines EDS and wT-metaD simulations was employed to explore the mechanism and compute Gibbs free energy barriers for the activation of the Abl1 kinase. This strategy has two distinct advantages. First, by employing the EDS path as a CV, it alleviates the problem of choosing appropriate geometric CVs to describe the activation. Second, it allows for the possibility of exploring activation pathways that could be more favorable than the input EDS path. Through this strategy, we identified a three-step mechanism for the activation, wherein a cooperative effect was observed between structural changes in the A-loop and the DFG motif. The mechanism involves two intermediates and has a rate-determining free energy barrier of 13.8 kcal/mol. Both of the intermediates possessed a partially unfolded A-loop, differ primarily in the “in” or “out” conformation of the DFG motif, and are separated by a free energy barrier of 7.5 kcal/mol. Among the two, the DFG-in intermediate resembles the inactive state of src-kinase. The two intermediates may serve as attractive drug targets in future treatments of CML. Furthermore, exploring the effect of phosphorylation on the activation of Abl1, we have noted that the proposed strategy could not capture the DFG-flip for the phos system and overestimates the activation barriers. While the former observation can be associated with limitations in the EDS subspace in recovering small amplitude collective motions in the DFG flip, the latter may be due to insufficient sampling of structural changes triggered by phosphorylation of the inactive state.

## Supporting Information Available

Backbone rmsd between the two intermediates and the EDS path for inactivation, errors in estimated free energies, variation in rmsd_active_ during four different EDS simulations, Cα rmsd between consecutive frames sampled from EDS, Cα rmsd_active_ for the frames sampled from EDS, MFEP for the activation of the phos system, Ramachandran angles of Asp^381^ of the DFG motif, variation in rmsd_active_, the Ramachandran angles, the A-loop SASA, and the number of hydrogen bonds during the MFEP for the phos system (PDF)
mfep-profile-np.ipynb: a jupyter notebook with python code for extracting MFEP from the wT-metaD simulations of the non-phos system, mfep-traj-np.ipynb: a jupyter notebook with python code for generating a trajectory of MFEP from the wT-metaD simulations of the non-phos system, and block-average-np.ipynb: a jupyter notebook with python code and bash script for estimating the error in MFEP by means of block averaging from the wT-metaD simulations of the non-phos system (ZIP)
MFEP for the non-phos system (MPG)
MFEP for the phos system (MPG)

## Supplementary Material

ct1c00505_si_002.zip

ct1c00505_si_003.mpg

ct1c00505_si_004.mpg
