# Supplementary material for: Activation of Abl1 Kinase Explored Using Well-Tempered Metadynamics Simulations on an Essential Dynamics Sampled Path
Source: J Chem Theory Comput. 2021 Oct 14;17(11):7260–70. doi: 10.1021/acs.jctc.1c00505 (PMC8582261; doi:10.1021/acs.jctc.1c00505)
Supplement: Supplementary file 1 — ct1c00505_si_001.pdf [file ct1c00505_si_001.pdf]

Activation of Abl1 Kinase Explored using Well-Tempered Metadynamics Simulations on an Essential Dynamics Sampled Path Supporting Information

Baswanth Oruganti and Ran Friedman*

Department of Chemistry and Biomedical Sciences, Faculty of Health and Life Sciences, Linnæus University, 391 82 Kalmar, Sweden

E-mail: ran.friedman@lnu.se

Table S1: Full backbone RMSD (in Å) of the two intermediate structures in the proposed mechanism with respect to the corresponding Abl1 conformations reported previously by Meng *et al.*[a]

Conformation ID	RMSD of intermediate-1 (bin 11)	RMSD of intermediate-2 (bin 15)
1[b]	3.56	**2.53**
2	3.73	2.74
3	3.33	4.57
4[b]	3.54	**2.32**
5	3.59	4.68
6	3.76	2.61
7[c]	**2.77**	3.45
8	3.93	3.23
9[c]	**2.71**	3.22
10	3.61	3.28
11	3.33	3.28
12	4.25	5.55
13	3.63	2.61
14[c]	**2.81**	3.74
15	3.67	4.98
16	3.76	2.71

[a] See Reference 11 of the main manuscript. The backbone atoms of the two intermediates are aligned with those of the confromations reported in the Reference 11 before computing RMSD values.

[b] Conformations proximate to intermediate-2.

[c] Conformations proximate to intermediate-1.

Table S2: Converged errors (kcal/mol) in the estimated free energies of MFEP configurations for the non-phos and phos systems.

Bin index	non-phos	phos
1	0.2	0.5
2	0.2	0.3
3	0.2	0.4
4	0.2	0.4
5	0.2	0.4
6	0.3	0.4
7	0.4	0.3
8	0.4	0.4
9	0.4	0.4
10	0.4	0.4
11	0.5	0.5
12	0.5	0.5
13	0.4	0.4
14	0.4	0.3
15	0.4	0.3
16	0.3	0.3
17	0.4	0.3
18	0.3	0.3
19	0.3	0.3
20	0.2	0.3
21	0.3	0.3
22	0.3	0.3
23	0.3	0.4
24	0.3	0.5
25	0.4	0.4

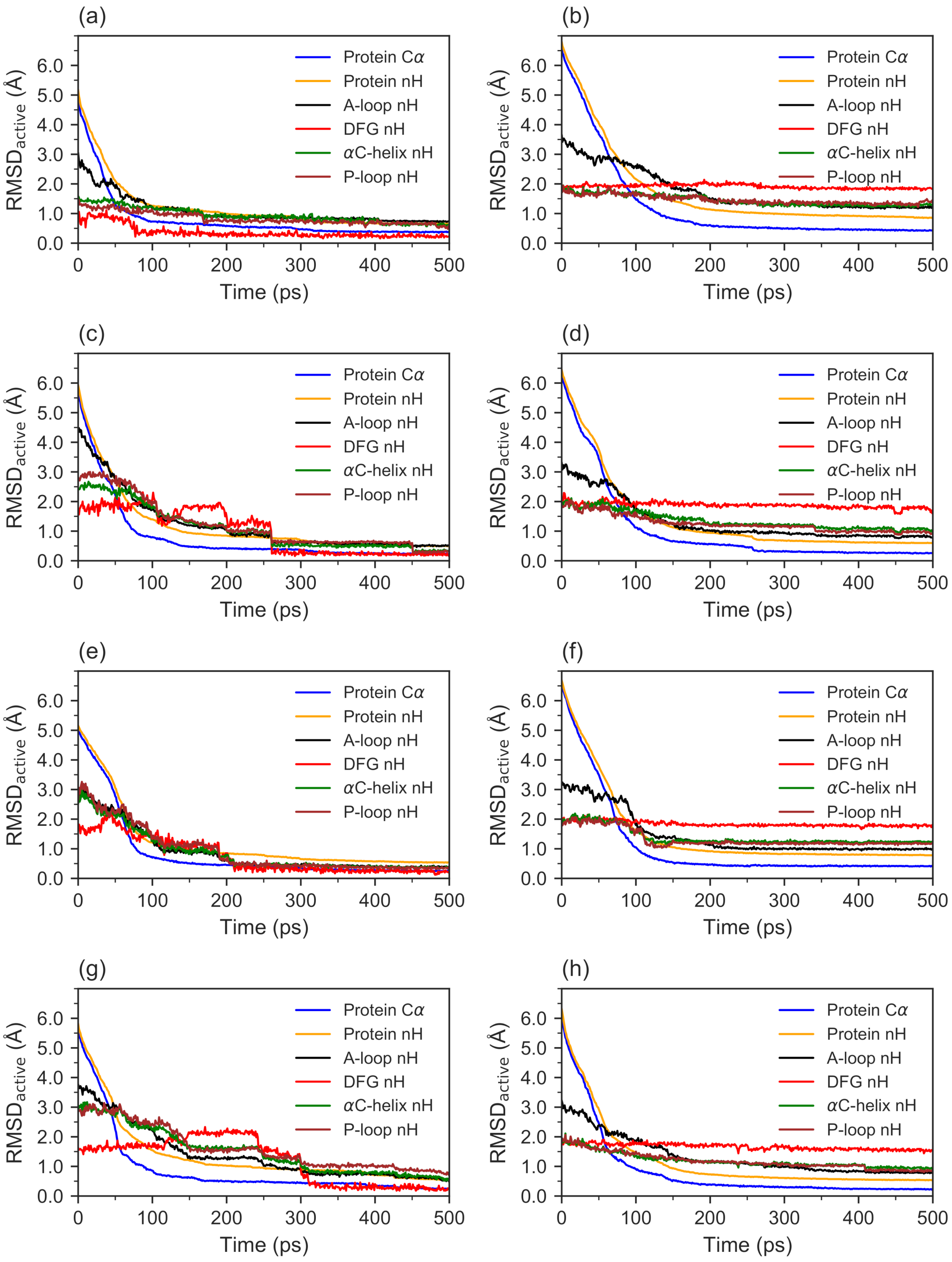

Figure S1: Variation in RMSD relative to the target active state (RMSD$_{active}$) of the non-phos (left panel: (a), (c), (e), (g)) and the phos (right panel: (b), (d), (f), (h)) systems during four different EDS simulations. Before each type of RMSD calculation, EDS structures were fitted to the active state using either the Cα (for Cα-RMSD) or non-hydrogen (for nH-RMSD) atoms of only the structural element under consideration.

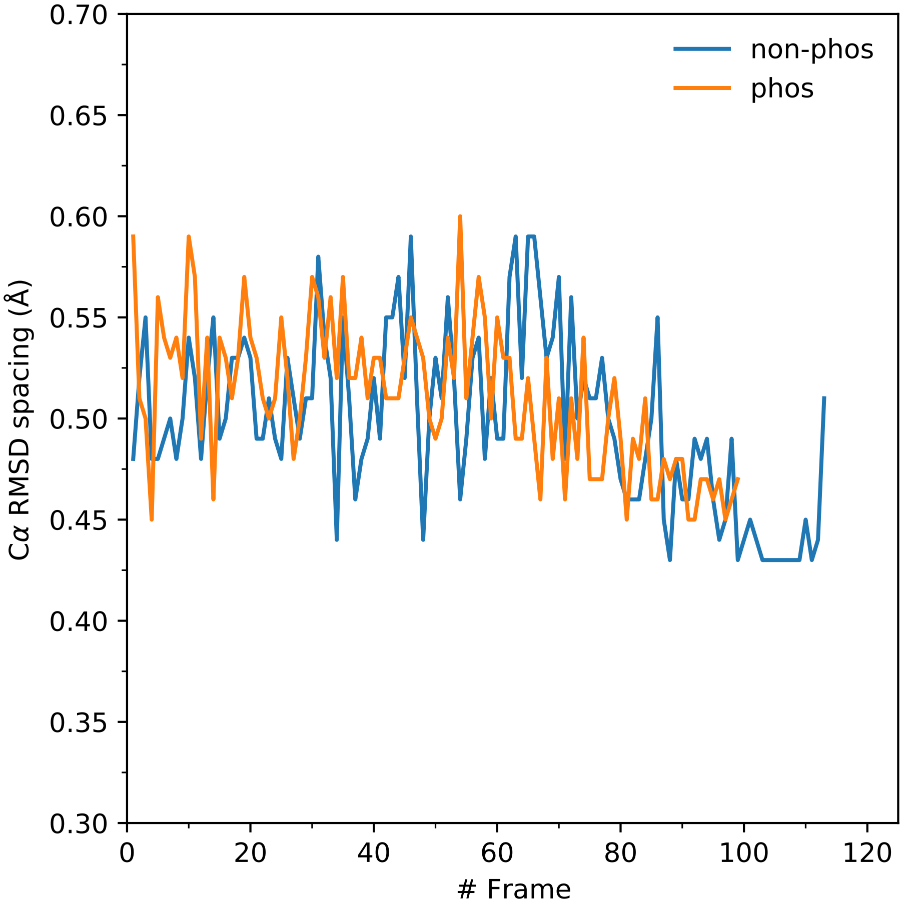

Figure S2: Cα RMSD spacing between the consective frames of the non-phos and the phos systems sampled from EDS. Note that each label on the X-axis represent the first frame index of the two consecutive frames.

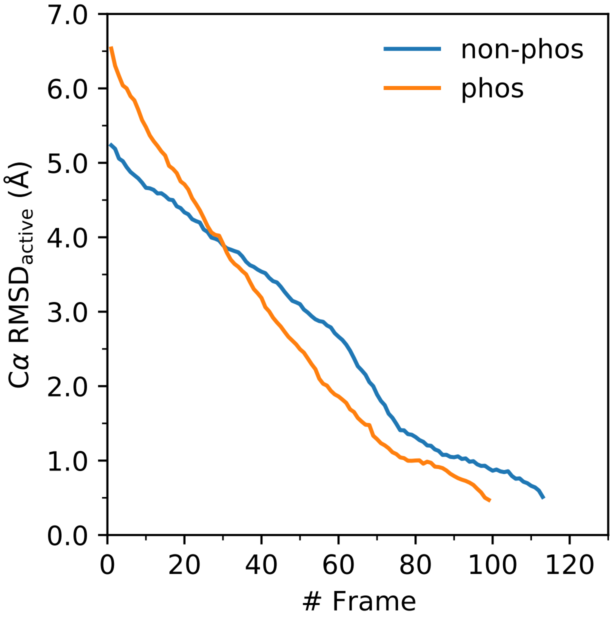

Figure S3: Variation in Cα RMSD relative to the target active state (RMSD$_{\text{active}}$) of the non-phos and the phos systems for the frames sampled from EDS.

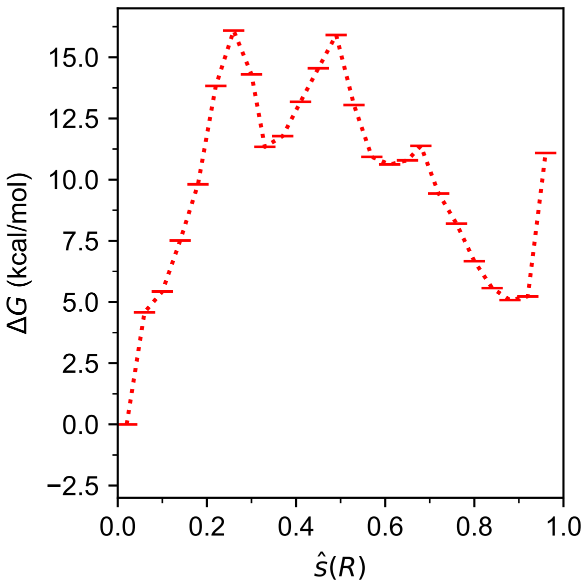

Figure S4: MFEP for activation of the phos system as a function of the $\hat{s}(R)$ CV obtained from the wT-metaD simulations.

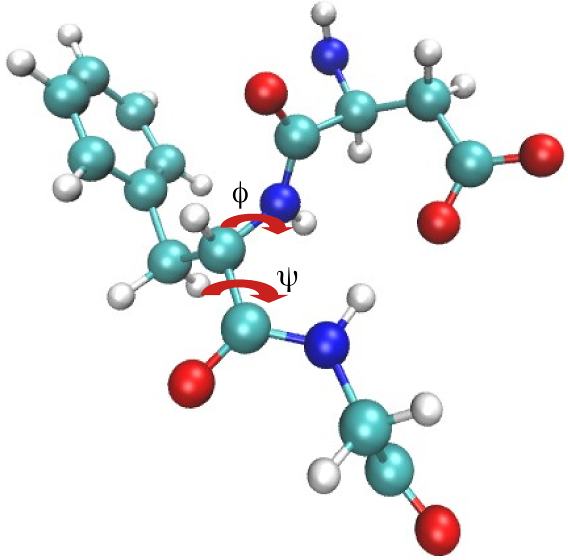

Figure S5: Ramachandran angles ψ and ϕ of Phe^{382} of the DFG motif.

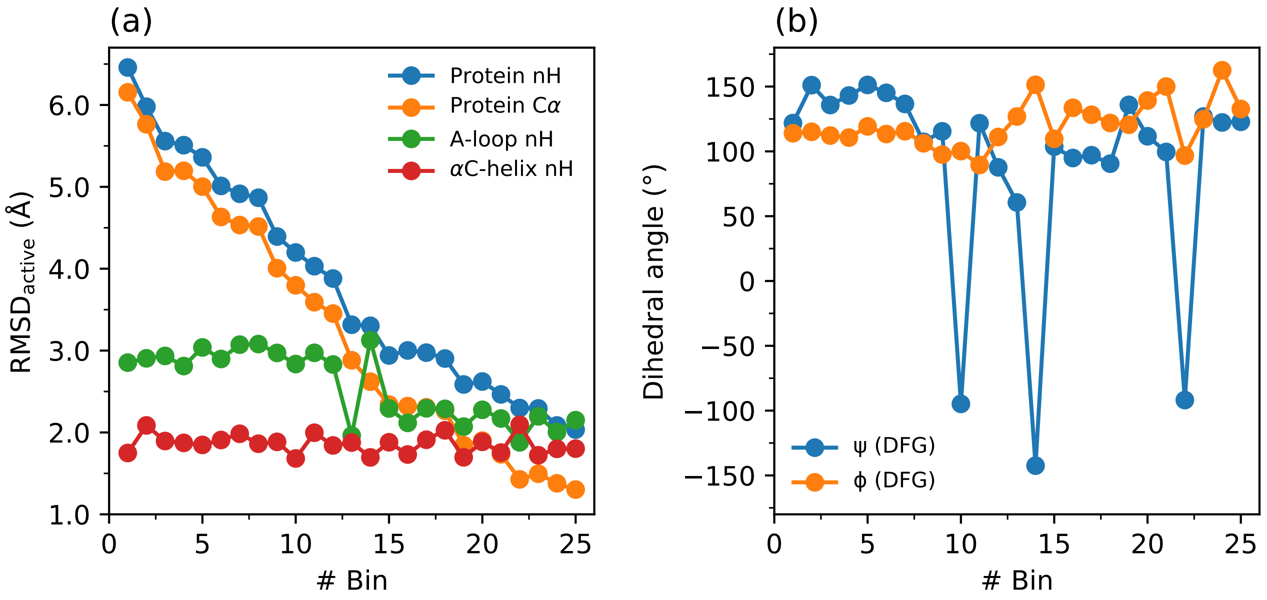

Figure S6: Variation in RMSD with respect to the target active state (RMSD$_{\text{active}}$) (a), and variation in the Ramachandran angles ψ and ϕ (b) during the MFEP of the phos system.

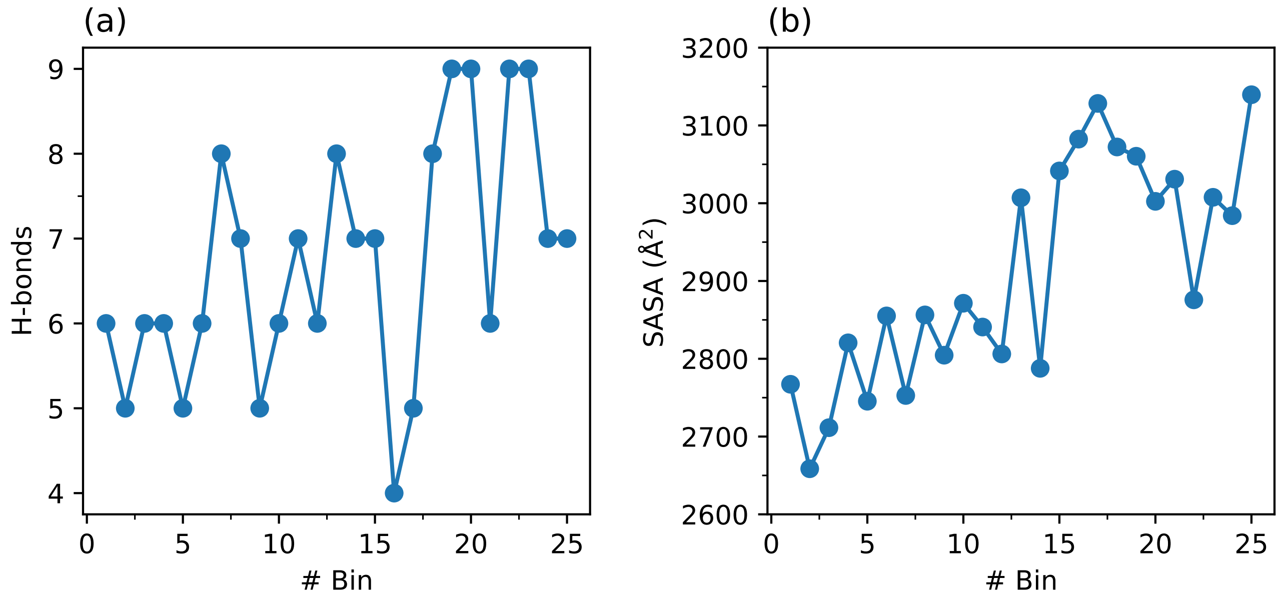

Figure S7: Variation in the A-loop SASA (a), and variation in the number of hydogen bonds between the A-loop and the solvent (b) during the MFEP of the phos system.
